# When your brain looks older than expected: combined lifestyle risk and BrainAGE

**DOI:** 10.1007/s00429-020-02184-6

**Published:** 2021-01-10

**Authors:** Nora Bittner, Christiane Jockwitz, Katja Franke, Christian Gaser, Susanne Moebus, Ute J. Bayen, Katrin Amunts, Svenja Caspers

**Affiliations:** 1 Institute for Anatomy I, Medical Faculty, Heinrich-Heine University Düsseldorf, Universitätstr. 1, 40225 Düsseldorf, Germany; 2 Institute of Neuroscience and Medicine (INM-1), Research Centre Jülich, 52425 Jülich, Germany; 3 Structural Brain Mapping Group, University Hospital Jena, 07743 Jena, Germany; 4 Institute of Urban Public Health, University of Duisburg-Essen, 45122 Essen, Germany; 5 Mathematical and Cognitive Psychology, Institute for Experimental Psychology, Heinrich-Heine University Düsseldorf, 40225 Düsseldorf, Germany; 6 Cecile and Oskar Vogt Institute for Brain Research, Medical Faculty, Heinrich-Heine University Düsseldorf, 40225 Düsseldorf, Germany; 7 JARA-BRAIN, Juelich-Aachen Research Alliance, 52425 Jülich, Germany

**Keywords:** MR-morphometry, MR-based age estimation, Lifestyle, Smoking, Physical activity

## Abstract

Lifestyle may be one source of unexplained variance in the great interindividual variability of the brain in age-related structural differences. While physical and social activity may protect against structural decline, other lifestyle behaviors may be accelerating factors. We examined whether riskier lifestyle correlates with accelerated brain aging using the BrainAGE score in 622 older adults from the 1000BRAINS cohort. Lifestyle was measured using a combined lifestyle risk score, composed of risk (smoking, alcohol intake) and protective variables (social integration and physical activity). We estimated individual BrainAGE from T1-weighted MRI data indicating accelerated brain atrophy by higher values. Then, the effect of combined lifestyle risk and individual lifestyle variables was regressed against BrainAGE. One unit increase in combined lifestyle risk predicted 5.04 months of additional BrainAGE. This prediction was driven by smoking (0.6 additional months of BrainAGE per pack-year) and physical activity (0.55 less months in BrainAGE per metabolic equivalent). Stratification by sex revealed a stronger association between physical activity and BrainAGE in males than females. Overall, our observations may be helpful with regard to lifestyle-related tailored prevention measures that slow changes in brain structure in older adults.

**Supplementary Information:**

The online version contains supplementary material available at 10.1007/s00429-020-02184-6.

## Introduction

Structural brain changes during normal aging comprise decreases in gray matter (GM) and white matter (WM; Fjell and Walhovd 2010). Interestingly, some older individuals experience strong and early manifestations (accelerated brain aging), while others of comparable age do not experience changes expected at that age [decelerated brain aging; (Bartrés-Faz and Arenaza-Urquijo 2011; Ziegler et al. 2012)]. As this high interindividual variability cannot be fully explained by chronological age (Jockwitz et al. 2017; Stern 2009, 2012), other factors that provide potential explanatory insight have come into focus, one of them being lifestyle.

According to the seed and soil model (McDonough and Allen 2019) neurocognitive disorders are only developed, if pathological processes such as cell death and accumulation of neurofibrillary tangles (the seed) meet an unfavorable neuronal environment (the soil). Unfavorable neuronal environments can be health conditions accompanying normal aging such as cardiovascular disease or infections, but also behavior such as a risky lifestyle (McDonough and Allen 2019). In contrast, a protective environment, e.g., healthier lifestyle such as higher physical activity and social integration, may promote a more resilient soil and neuroprotection (Anaturk et al. 2018; Arenaza-Urquijo et al. 2015; Bittner et al. 2019; Fratiglioni et al. 2004; McDonough and Allen 2019). For example, socially integrated Alzheimer’s disease patients show higher cognitive stability compared to not integrated patients, even when suffering from a similar degree of pathology (Bennett et al. 2006). Furthermore, social network size correlates positively with amygdala volume in humans (Bickart et al. 2011). Likewise, higher physical activity, especially in older adults, has repeatedly been associated with better cognitive performance (Colcombe and Kramer 2003; Erickson et al. 2007; Hughes and Ganguli 2009; Kramer et al. 1999, 2003; Voelcker-Rehage et al. 2010) and preservation of GM volume (Colcombe et al. 2003; Erickson et al. 2011). Older adults engaging in physical activity training showed increased hippocampal volume (Erickson et al. 2011) and more efficient use of functional brain networks (Colcombe et al. 2004; Voelcker-Rehage et al. 2011). Recently, lifespan physical activity has been associated with favorable ratios of brain metabolism markers in magnetic resonance imaging (Engeroff et al. 2019). This hints even further at physical activity being a factor for promoting a more resilient neuronal environment. In contrast, smoking seems to be associated with cortical thinning in prefrontal and temporal regions (Karama et al. 2015) and decreased GM density within cingulum, precuneus, thalamus, and precentral gyrus (Almeida et al. 2008). In addition, excessive alcohol consumption can lead to serious neurological diseases, e.g., Korsakow syndrome (de la Monte and Kril 2014) and is associated with reduced GM and WM volume and density (Paul et al. 2008; Pfefferbaum et al. 1995; Topiwala et al. 2017) in alcohol-dependent as well as non-dependent individuals (Mukamal at al. 2001).

Most previous studies focused on specific effects of a single lifestyle variable on brain structure and function. In real life, however, individuals engage in a combination of lifestyle behaviors, e.g., physical exercise and afterwards meeting friends (social integration) while drinking a beer (alcohol consumption). Only few studies investigated the effects of lifestyle on brain structure and function or on cognition in a multidimensional way. For example, Floel et al. (2008) found that the combination of exercise, dietary habits, BMI, smoking and alcohol intake was a better predictor for memory performance than the individual lifestyle behaviors. In a previous study, we developed a combined lifestyle risk score reflecting individual combinations of the above described daily lifestyle behaviors, with higher values reflecting more risky behavior (e.g., high smoking and alcohol consumption, low social integration and physical activity), whereas lower values indicate protective combinations (Bittner et al. 2019). We showed that higher combined lifestyle risk was associated with brain atrophy, e.g., more alcohol consumption combined with low physical activity was associated with structural decreases in the premotor region. From the perspective of the seed and soil theory, it may further be concluded that combination of several risky lifestyle factors may provide an even more unfavorable soil for the development of alterations in brain structure than the presence of one factor alone. Based on these findings, it might thus be assumed that combined risky lifestyle leads to accelerated brain aging, accompanied by decreases in cognitive performance. Non-linear effects and covariates such as sex or education as these affect not only brain phenotypes, but also lifestyle habits and the association between both are of additional relevance (Cullen et al. 2012; Fratiglioni et al. 2004; Gur and Gur 2017; Kramer and Colcombe 2018; McKenna et al. 2003; Mukamal et al. 2001).

However, we still do not know enough about which factors or behaviors predict the size of the gap between a specific chronological age and the actual manifestation of the individual aging process. To capture this manifestation, biological age of the brain, estimated from structural brain scans, may be more informative and precise than chronological age. To measure these MRI-based brain-aging patterns, Franke et al. (2010) developed a machine-learning framework, which uses the most relevant voxel-wise GM information to aggregate the complex underlying multidimensional alteration patterns of brain aging into one single value, the estimated brain age. The difference, i.e., the gap, between brain age as estimated from MR images and true chronological age (Franke et al. 2010) is then the Brain **A**ge **G**ap **E**stimation (BrainAGE) score. BrainAGE is positive if aging patterns observed via MRI appear older than expected based on chronological age (accelerated brain aging), and negative if they appear younger (decelerated brain aging). BrainAGE and several other MRI-based brain age prediction models have been established as a meaningful imaging biomarker to study the prediction of future brain aging patterns and their longitudinal trajectories (Cole and Franke 2017; Cole et al. 2019; Franke et al. 2012; Gaser et al. 2013), cognitive decline and disease severity (Franke et al. 2012). At the same time, BrainAGE is widely applicable and highly accurate to study the high interindividual variability in structural brain aging (Cole and Franke 2017). Recent studies found MRI-predicted brain age even to be associated with relevant genetic variants for cortical thickness and encoding for tau protein (Jónsson et al. 2019; Ning et al. 2020), even though these variants only explained 11% of the variance at most within the gap between estimated brain and chronological age. In contrast, other brain age predictions have been reported to be not associated to genetic variants in general, but only specific brain compartments, which in turn were partly related to modifiable risk behavior, e.g., smoking (Smith et al. 2020).

Hence, the current study aimed at examining whether highly complex, multivariate lifestyle behaviors can partly predict the deceleration or acceleration of brain aging (reflected in the BrainAGE score) in the 1000BRAINS cohort of “normal” aging older adults (Caspers et al. 2014). Here, BrainAGE allows conducting straightforward quantification of how much variance in brain aging can be predicted by a more risky lifestyle, and therefore, whether it may be a potential soil for neurocognitive alterations (McDonough and Allen 2019).

First, we examined the relation between our newly developed combined lifestyle risk score (Bittner et al. 2019) and BrainAGE. We hypothesized that higher combined lifestyle risk would generally predict accelerated brain aging, i.e., higher BrainAGE scores. Second, we investigated the association between each individual lifestyle variable and BrainAGE to further elucidate contributions of single lifestyle variables to this age acceleration prediction. We ran all analyses for the whole sample and subsequently for males and females separately (based on separate BrainAGE estimations) to account for sex-specific differences.

## Materials and methods

### Participants

One thousand, three hundred and sixteen participants with an age range from 18.5 to 87.0 years were available from the 1000BRAINS study (Caspers et al. 2014) of European ancestry. Due to the population-based nature of the 1000BRAINS study, the only exclusion criteria were contraindications for the MR session (Caspers et al. 2014). From the overall cohort sample, 87 participants were exluded due to missing MR scans, methodological failure during data processing (see MRI preprocessing) or missing BrainAGE estimation. Hence, 1229 MR datasets were available as training sample for brain age estimation (see “Age Estimation Framework”).

For the lifestyle analyses, we used data of those participants aged older than 55 years recruited from the Heinz Nixdorf Recall study (Schmermund et al. 2002). From those 1229 participants with available MR data, *n* = 666 were within the selected age range. Two participants had to be excluded from the lifestyle analyses due to incidental findings, and 42 participants due to missing values in behavioral data. Finally, the older subsample for the lifestyle analyses consisted of 622 participants (272 females, 350 males) with an age range of 56–85 years (mean = 67.5 years, SD = 6.7). The study protocol of 1000BRAINS was approved by the Ethics Committee of the University of Essen (Germany). All participants gave written informed consent in agreement with the declaration of Helsinki.

### Lifestyle measures

Lifestyle data were retrieved from the database of the third examination (10-year follow-up) of the Heinz Nixdorf Recall study that commenced in June 2011 (Caspers et al. 2014; Schmermund et al. 2002).

#### Alcohol consumption

Average consumption of different alcoholic beverages (beer as 0.2 l, red and white wine as one glass of 0.2 l, and spirits as 0.02 l) within the last 4 weeks was assessed via a self-report questionnaire (Schmermund et al. 2002). The proportion of pure alcohol within the specific beverage was then multiplied with the frequency of drinking. Next, all beverages per person were summed up, resulting in the total amount of pure alcohol consumption in grams per month (g/month). Alcohol consumption as assessed via self-report questionnaires has been shown to highly correlate with blood indices of alcohol consumption (Glovannucci et al. 1991), multiple weekly self-report diet records (Glovannucci et al. 1991) and transdermal alcohol-use assessment (Simons et al. 2015), thus providing adequate reliability and validity for most research purposes (Del Boca and Darkes 2003).

#### Smoking

The degree of lifetime exposure to tobacco smoking was assessed as pack-years (Duriez et al. 2014; Franklin et al. 2014; Karama et al. 2015), calculated by multiplying the years of smoking with the self-reported number of smoked cigarettes per day.

#### Social integration

Social integration was assessed using an adapted version of the social integration index (Berkman 2004). The present social integration index comprised the domains “Marital status” (married or cohabitating participants were scored a 2; single, never married, widowed, or divorced participants were scored a 0), “close ties” (sum score derived from the number of children, close relatives, and friends) and “membership in organizations” (sum score of the number of organizations participants were members in and participated in at least once a month). Organizations included were: sport clubs, regional clubs, hunting clubs, choirs, theater clubs, music clubs, occupational or labor unions, political clubs or parties, congregations, and self-help groups. The scores of all three domains were summed up into the social integration score.

#### Physical activity

To measure physical activity, we used the metabolic equivalent of task (MET; Ainsworth et al. (2000) measuring the energy expenditure of a given activity compared to rest. The compendium of physical activities (Ainsworth et al. 2000) provides a mean energy expenditure value per hour of each activity. Participants were asked to report up to four different sportive activities, carried out within the last month. Based on the MET values assigned to the activities listed in the compendium, MET values were assigned to each of the activities reported by the participants and multiplied by the duration in hours (per month). Finally, a sum score of all activities was calculated. In addition, body mass index (BMI) was measured, since it has been shown to affect the association between physical activity and brain volume (Ho et al. 2011).

#### Construction of the combined lifestyle risk score

The combined lifestyle risk score was constructed as used in a previous study (Bittner et al. 2019). That is, to standardize the measurements of the four lifestyle variables, we first transformed the raw score on each individual lifestyle variable into a *z*-score. Next, to obtain a risk score that indicated higher risk with higher values, we reversed signs of the protective behaviors (social integration and physical activity). To obtain a risk score where a value of zero would indicate a mathematical balance of negative and protective behaviors, an additional linear transformation of the *z*-transformed lifestyle behaviors was applied: The protective variables were linearly transformed into negative scores by subtracting the maximum value from each individual measurement, whereas risk behaviors were analogously transformed into positive scores by adding the minimum value to each individual measurement. Hence, all values for risk behaviors were positive. Finally, the linearly transformed values of all individual lifestyle variables were summed up into one combined lifestyle risk score.

#### Covariates

Since we aimed at investigating the residual variability in brain aging, we considered chronological age to be a covariate as it was associated to the BrainAGE score (Fig. 2b). However, it is not conclusive, whether chronological age should be included within statistical analyses on BrainAGE, since BrainAGE is based on chronological age (Smith et al. 2019). We, therefore, conducted three different approaches to address this issue: approach 1 included chronological age as a predictor in the regression models of lifestyle on BrainAGE, approach 2 did not include chronological age within the models and for approach 3, chronological age was regressed out of BrainAGE, before using BrainAGE as a dependent variable.

The brains of women and men show differences with respect to their structural architecture, e.g., in the proportion of gray matter and the thickness of the cortex (Ritchie et al. 2018), as well as aging trajectories (Cosgrove et al. 2007; Franke et al. 2014; Gur and Gur 2017; Ruigrok et al. 2014; Wierenga et al. 2018), functional network organization sub-serving cognitive abilities (Jiang et al. 2020), as well as the morphology of the so-called social brain (Kiesow et al. 2020). Hence, we considered sex as a covariate in the statistical models, and separately examined sex differences in performance measures and lifestyle analyses.

In addition, higher education is associated with higher cognitive performance (Elias et al. 1997) and is generally considered a proxy for brain reserve, the ability to better tolerate age-related neuronal loss (Bartrés-Faz and Arenaza-Urquijo 2011; Christensen et al. 2008; Stern 2012). Furthermore, there may be associations between education, intelligence, and lifestyle behavior (Cullen et al. 2012; Fratiglioni et al. 2004), where less smoking has been found in more educated individuals (McKenna et al. 2003). Hence, we hypothesized general educational level as a possible confounding factor and added education as a covariate into the statistical model. General education was measured using the international standard classification of education (UNESCO 1997), a standard classification system with 10 levels, where higher levels indicate higher education. Further, cognitive, physical and mental health of our older participants was estimated. Physical and mental well-being was estimated using the SF36 self-report questionnaire (SF-36 Fragebogen zum Gesundheitszustand (Franke et al. 1998) assessing quality of life in relation to health. As body mass index (BMI) has been shown to influence the association between physical activity and brain structure (Ho et al. 2011) and memory performance (Floel et al. 2008), it was considered as a covariate in the analyses of the combined lifestyle risk score, as well as of physical activity. Cognitive performance was examined using the DemTect test (Kalbe et al. 2004), a screening tool for dementia symptoms including verbal and working memory, word fluency and intellectual flexibility examinations. The DemTect test was designed to provide information on the general cognitive status. The present study used these estimates, i.e., physical and mental well-being, as well as cognitive status, to provide more information on the general fitness of the here presented older adult sample.

### MRI preprocessing

T1-weighted anatomical 3D images were collected with a 3 T Tim-TRIO MR scanner (Siemens Medical System, Erlangen, Germany). The following scan parameters were used: TR = 2.25 s, TE = 3.03 ms, TI = 900 ms, FoV = 256 × 256 mm^2^, flip angle = 9°, voxel resolution = 1 × 1 × 1 mm^3^, and 176 axial slices. A detailed description of the 1000BRAINS study protocol can be found in Caspers et al. (2014). MRI preprocessing was done using the SPM12 toolbox (The Wellcome Dept. of Imaging Neuroscience, London; www.fil.ion.ucl.ac.uk/spm) and the CAT12 package (http://dbm.neuo.uni-jena.de) running under Matlab (The MathWorks Inc., Natick, MA, USA). First, T1-weighted images were corrected for bias field inhomogenities and spatially normalized. Then, images were segmented (Ashburner and Friston 2005) into gray matter (GM), white matter (WM) and cerebro-spinal fluid (CSF) using an approach additionally accounting for partial volume effects (Tohka et al. 2004) by applying adaptive maximum a posteriori estimations (Rajapakse et al. 1997) and a hidden Markov Random Field Model (Cuadra et al. 2005), as described in Franke et al. (2010). From these segmentation maps, only GM maps were used for the BrainAGE estimation framework. GM maps were registered using an affine registration and further smoothed with an 8-mm full-width-at-half-maximum (FWHM) kernel, while resampling the volumes at 8-mm spatial resolution. Next, since neighboring voxels are spatially correlated and therefore contain redundant information, principal component analysis (PCA) was conducted to reduce data dimensions using the “Matlab toolbox for Dimensionality Reduction” (version 0.7b; Van der Maaten (2007).

### Age estimation framework

#### Training data

**Fig. 1:**
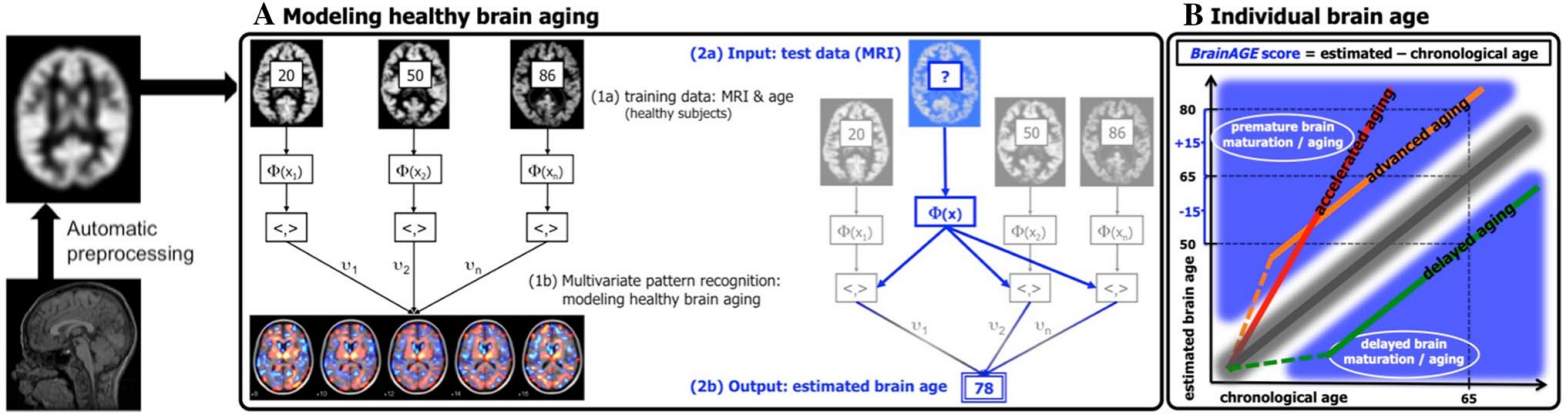
Representation of the BrainAGE concept. **a** The model of healthy brain aging is trained with the chronological age and preprocessed structural MRI data of a training sample (left, with an exemplary illustration of the most important voxel locations that were used by the age regression model). Subsequently, the individual brain ages of previously unseen test subjects are estimated, based on their MRI data (blue, picture modified from Schölkopf et al. 2002). **b** The difference between the estimated and chronological age results in the BrainAGE score. Consequently, positive BrainAGE scores indicate accelerated brain aging (Franke et al. 2010) [Figure modified from Franke et al. (2012)]

The BrainAGE framework is based on a relevance vector machine (RVM) (Tipping 2001) that transforms training data into a high-dimensional space (Bennett and Campbell 2000) and translates features learned from a training sample for a specific outcome variable (i.e., age) onto an unkown test sample (Fig. 1). To train and test the BrainAGE framework with respect to prediction accuracy and reliability, we used a ‘leave 10 out’-procedure within the full cohort of 1000BRAINS, which spans 1,229 MR datasets with an age range from 18.5 to 87.0 years (*M* = 60.7 years, SD = 13.4 years; *M*_male_ = 60.7 years, SD_male_ = 14.0 years; *M*_female_ = 60.9 years, SD_female_ = 12.5 years). Even though our study focuses on the association between lifestyle behavior and brain aging in older adults, we chose to train the RVM on the full cohort of 1000BRAINS for two reasons: (i) from a neuropsychological perspective, brain aging is a highly complex phenomenon, which can - as a pattern - better be learned by the algorithm when having access to a larger age range, which in the full cohort spans the entire adult life span (18.5–87 years), i.e., the algorithm has access to phenotypically richer data. However, using the whole age range has methodological advantages as well (ii): having as many and as morphologically rich training data as possible within the learning phase results in higher (and therefore, more constant) degrees of freedom, higher predictive power, and higher accuracy. This has been shown even though training and test samples differ demographically [(Cole et al. 2015; Franke et al. 2010; Varikuti et al. 2018); for a recent review see Cole et al. 2019]. In the training stage, the input data (i.e., *n* - 10 of the whole sex-split sample) were used to train the BrainAGE framework using chronological age and the GM tissue probability maps (Franke et al. 2010). The algorithm was trained seperately for female and male participants, considering that different features may be relevant for age prediction in females and males, as has been shown for intelligence prediction (Jiang et al. 2020) and for social characteristics (Kiesow et al. 2020). Blind to their true chronological age, the patterns learned by the relevance vector regression were then transferred to the unknown 10 test participants that were left out during the training stage, such that estimated age was based only on their anatomical patterns within the GM maps. This procedure was repeated until estimated age was provided for the full cohort of 1000BRAINS. For each participant, true chronological age was then subtracted from estimated age:

$${\text{BrainAGE}} = {\text{estimated age}}-{\text{chronological age}}{.}$$

Positive BrainAGE scores reflect accelerated aging, i.e., the estimated age is higher than the chronological age. In contrast, negative BrainAGE scores reflect decelerated aging, i.e., the estimated age is lower than the chronological age. Finally, a correction for a quadratic age trend, which is identifiable in Fig. 1a, was applied to the resulting BrainAGE values using spm_detrend (SPM12, The Wellcome Dept. of Imaging Neuroscience, London; www.fil.ion.ucl.ac.uk/spm).

### Statistical analysis

In the first step, we explored performance measures of the BrainAGE framework. In a second step, we investigated the influence of possible covariates (sex and education) on BrainAGE to examine whether we need to consider their influences in the lifestyle analyses. In the main analyses, we then examined the assocations between combined lifestyle risk, as well as the individual lifestyle behaviors and BrainAGE. All statistical analyses were carried out using IBM SPSS Statistics 26.0.

#### Performance measures for the BrainAGE estimation

Following recent recommendations on age estimation models (Cole and Franke 2017) and the machine-learning literature (Bzdok and Ioannidis 2019; Yarkoni and Westfall 2017), we provide study sample-specific performance measures for the age estimation framework applied within this study. To test the accuracy of our model, we took the mathematical absolute value of each BrainAGE value and calculated the sample mean, which should then reflect the mean absolute error (MAE) of the brain age estimation. The smaller the MAE is, the better the performance of the estimation (Cole and Franke 2017). The idea behind is that each individual BrainAGE value can also be interpreted as the error of the age estimation model (i.e., the extent to which the estimation deviates from the true age). In addition, we examined Pearson correlations between estimated age, chronological age, and BrainAGE.

#### Relation between covariates and BrainAGE

We calculated a between-subjects multivariate analysis of co-variance (MANCOVA) with sex as independent factor (male vs female), educational level as covariate and chronological age, estimated age, and BrainAGE as dependent variables. Sex differences in educational level were examined using a between-subject ANCOVA with independent factor sex (male vs female), covariate chronological age and education as dependent variable. In addition, we calculated Spearman correlations (method of choice for ordinally scaled variables, such as the ISCED) between education and BrainAGE. Further, we analyzed descriptive statistics of physical and mental well-being, as well as congitive status of the older subsample for sample description.

#### Lifestyle and BrainAGE

In our main analyses, we investigated the associations between lifestyle and BrainAGE using a twofold approach. First, we examined whether and how well BrainAGE would be predictable by our combined lifestyle risk score and second, we examined the prediction of BrainAGE by each individual lifestyle variable.

To investigate the linear effect of combining the four lifestyle variables into one score, we calculated multiple linear regressions (with IBM SPSS Statistics 26.0). The initial model (model 1) analyzing combined lifestyle risk used the combined lifestyle risk score and sex as explanatory variables. The second analysis introduced sex and the individual lifestyle behaviors (physical activity, social integration, alcohol consumption, and pack-years of cigarettes) as explanatory variables in model 1. In both analyses we used BrainAGE as dependent variable to be predicted. In a second model for each analysis, we applied a post hoc outlier exclusion (values > 3 SD) for each variable that showed a significant effect on BrainAGE. As additional covariates education and cognitive status were added within a third and a forth model. Please note, that each analysis step, i.e., model, was carried out for each of the approaches 1, 2 and 3. To assess whether the combined lifestyle risk score explaines more variance than the individual lifestyle variables, we compared the explained variance in R^2^ of both approaches. Further we supplement our analyses with prediction metrics (mean absolute error, MAE) assessing how well the lifestyle models predict the individual expression of the BrainAGE score. MAE was calculated as the sum of the absolut value of the unstandardized residuals of each regression.

##### Sex differences in the association between lifestyle and BrainAGE

Subsequently, we wanted to know whether sex changes the association between lifestyle and BrainAGE. We calculated a between-subjects ANCOVA using *sex* as independent between-subjects factor, chronological age, education and the combined lifestyle risk score as covariates and BrainAGE as dependent variable, while introducing an interaction term between sex and the combined lifestyle risk score to test for a moderating effect of sex on the association between lifestyle risk and BrainAGE. To test for moderating effects of sex on the association between the individual lifestyle behaviors and BrainAGE, we calculated a between-subjects MANCOVA using the same independent factor, but using chronological age, education and social integration, physical activity, alcohol consumption, and smoking as covariates, while introducing interaction terms between sex and each respective lifestyle variable.

Next, we performed separate multiple linear regression analyses for the two sexes first without introducing covariates, then applying the oulier correction, then adding education and cognitive status (and BMI where necessary).

In sum, these were 15 main tests (four lifestyle variables, the combined lifestyle risk score, i.e., 5 variables tested, the main analysis in the whole subsample and the subsequent sex-stratified analyses) such that we compared the final results against a Bonferroni-adjusted *p* value of *p*_adjusted_ = 0.05/15 ≈ 0.003.

*Quadratic effects*: To further test for quadratic effects of the combined lifestyle risk score, as well as individual lifestyle behaviors on BrainAGE, we used curve fitting to compare whether quadratic functions result in a better fit for any lifestyle variable and BrainAGE by first regressing the linear effect of the three covariates age, sex and education out and then using the residuals of the respective lifestyle variable as input. The linear function was defined as:

$${\text{BrainAGE}} = {\text{intercept}} + b_{{{\text{1l}}}} x\;{\text{lifestyle}} + \varepsilon ,$$

whereas the quadratic function was defined as

$${\text{BrainAGE}} = {\text{intercept}} + b_{1q} x\;{\text{lifestyle}} + b_{2q} x\;^{2} + \varepsilon ,$$

subscript “l” indicates that the regressor belongs to the linear model, whereas subscript “q” indicates that the regressors belong to the quadratic function. In the final step, linear and quadratic functions were compared with regard to the explained variance R^2^.

##### Quantification of lifestyle effects

To quantify the effect of the combined lifestyle risk score and those individual lifestyle variables that showed a significant effect on BrainAGE, we estimated the slopes of the linear regression line for each explanatory factor using the linear equation:

$${\text{BrainAGE}} = {\text{intercept}} + b_{1} x\;{\text{sex}} + b_{2} x\;{\text{age}} + b_{3} x\;{\text{lifestyle}} + \varepsilon .$$

We used the intercept and unstandardized regression coefficients as calculated in the multiple linear regression as input for this equation, while the respective lifestyle variable (i.e., the combined lifestyle risk score or one individual lifestyle variable) was set to 1. We then multiplied the parameter *b*_3_ with 12 months. These “additional months of age” reflect the increase in months of BrainAGE with one increase in the explanatory variable, while the effect of the lifestyle variable was already adjusted for the covariates of age and gender.

We defined groups to further analyze the association between smoking and BrainAGE and matched never (pack-years = 0, 82 male, 82 female) to moderate (pack-years < 20, 96 males, 68 female) to severe smokers (pack-years ≥ 20, 55 females, 109 males) for age. We then calculated an ANCOVA using the factors group (never vs. moderate vs. severe) and sex, and the covariates age and education on the dependent variable BrainAGE.

##### Power analysis

As the effects of lifestyle on the brain as well as effects in large population-based samples are generally rather small, e.g., Miller et al. (2016); Yarkoni and Westfall (2017), power analyses using GPower (Faul et al. 2009; http://www.gpower.hhu.de/) were calculated for the applied linear regression models as recommended for studies with a given sample size (Faul et al. 2009). Power reflects the probability of rejecting false null hypotheses, in our case rejecting an association between lifestyle and BrainAGE which is truly not there. Here, type-I error level *α*, the sample size of the lifestyle analyses (*n* = 622 for the whole subsample) and the fully adjusted model number of predictors (age, sex, education, cognitive status and the respective lifestyle variable of interest) were used. Effect sizes were taken from previous studies on the association between lifestyle and the brain in older adults of comparable age (Bugg and Head 2011; Karama et al. 2015).

#### Data availability

The datasets generated and/or analyzed during the current study will be made available from the corresponding author to other scientists on request in anonymized format and according to data protection policy in the ethics agreement.

## Results

### Performance of the BrainAGE estimation framework

**Fig. 2:**
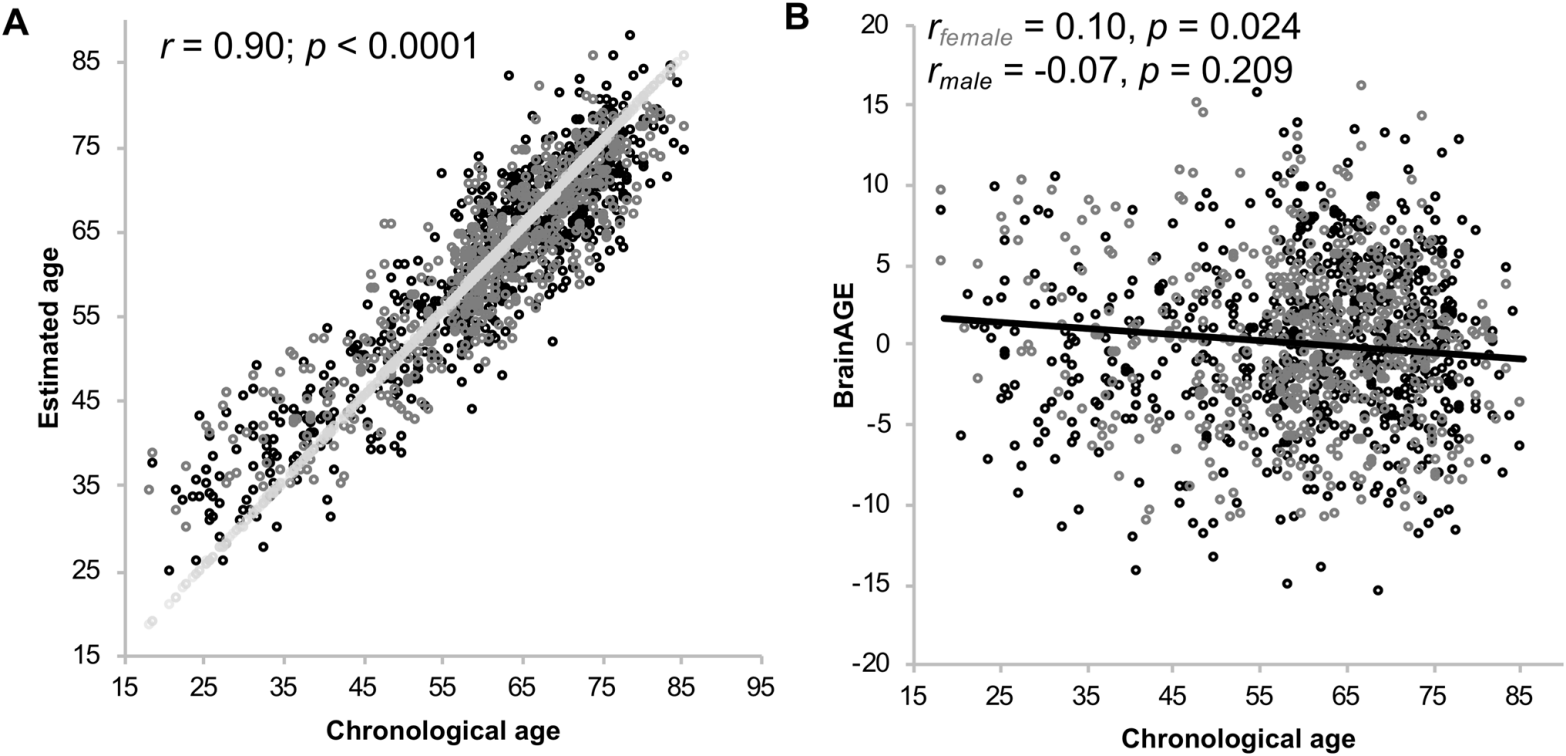
Scatter plots for the whole sample of 1000BRAINS, *n* = 1229. **a** Correlation between estimated and chronological age. Light-grey dots represent a regression line fitted to a simulated perfect correlation between estimated and chronological age of *r* = 1.0. **b** Correlation between chronological age and BrainAGE. The correlation was significant for female, but not for male participants. Black dots represent men, dark-grey dots represent women

Descriptive statistics of the training dataset, comprising the whole available sample of 1000BRAINS (*n* = 1229), are shown in Table 1. Mean BrainAGE was 0.00 (SD = 5.04). The mean absolute error (MAE) between chronological and estimated age was low with 4.62 years (SD = 3.67), respectively was the correlation very high (*r* = 0.90, *p* < 0.001, Table 2, Fig. 2a). The regression of estimated age on chronological age explained up to 83% of the variance (Table 1).

**Table 1:** Performance of the BrainAGE estimation framework

	Mean chronological age	Mean estimated age	Mean BrainAGE	MAE	*R*^2^
Whole sample of 1000BRAINS, *n* = 1229, age range 18.5–85.4					
Whole group	60.8 (13.4)	60.7 (11.6)	0.0 (5.0)	4.6 (3.7)	0.81
Male (*n* = 680)	60.7 (14.0)	60.8 (10.5)	0.0 (5.1)	4.6 (3.6)	0.78
Female (*n* = 549)	60.9 (12.5)	60.6 (12.4)	− 0.1 (4.9)	4.6 (3.7)	0.83
Older subsample used in analyses of lifestyle, *n* = 622, age range 56.2–85.4					
Whole group	67.5 (6.7)	66.8 (7.5)	− 0.2 (5.0)	4.0 (3.0)	0.51
Male (*n* = 350)	67.8 (6.7)	66.5 (7.2)	− 0.4 (5.1)	4.1 (3.1)	0.51
Female (*n* = 272)	67.1 (6.7)	65.7 (6.9)	0.1 (4.8)	3.8 (2.9)	0.52

Descriptive statistics and performance measures for the whole training sample, as well as the older subsample for lifestyle analyses. Age is given in years. Standard deviation is given in parentheses

*MAE* mean absolute error between estimated and chronological age, *R*^2^ explained variance drawn from linear regressions of estimated age on chronological age

**Table 2:** Correlations between chronological age, estimated age and BrainAGE

	Estimated age	BrainAGE
Whole sample of 1000BRAINS, *n* = 1229		
Chronological age		
Whole group	*r* = − 0.90, *p* < 0.001	*r* = 0.00, *p* = 0.999
Male (*n* = 650)	*r* = 0.92, *p* < 0.001	*r* = − 0.07, *p* = 0.083
Female (*n* = 549)	*r* = 0.88, p < 0.001	*r* = 0.10, *p* = 0.024
Older subsample used in analyses of lifestyle, *n* = 622		
Chronological age		
Whole group	*r* = 0.71, *p* < 0.001	*r* = − 0.10, *p* = 0.025
Male (*n* = 350)	*r* = 0.71, *p* < 0.001	*r* = 0.07, *p* = 0.209
Female (*n* = 272)	*r* = 0.72, *p* < 0.001	*r* =− 0.12, *p* = 0.047

Pearson correlations between chronological age, estimated age, and BrainAGE for the whole sample and the subsample used in the lifestyle analyses

**Fig. 3:**
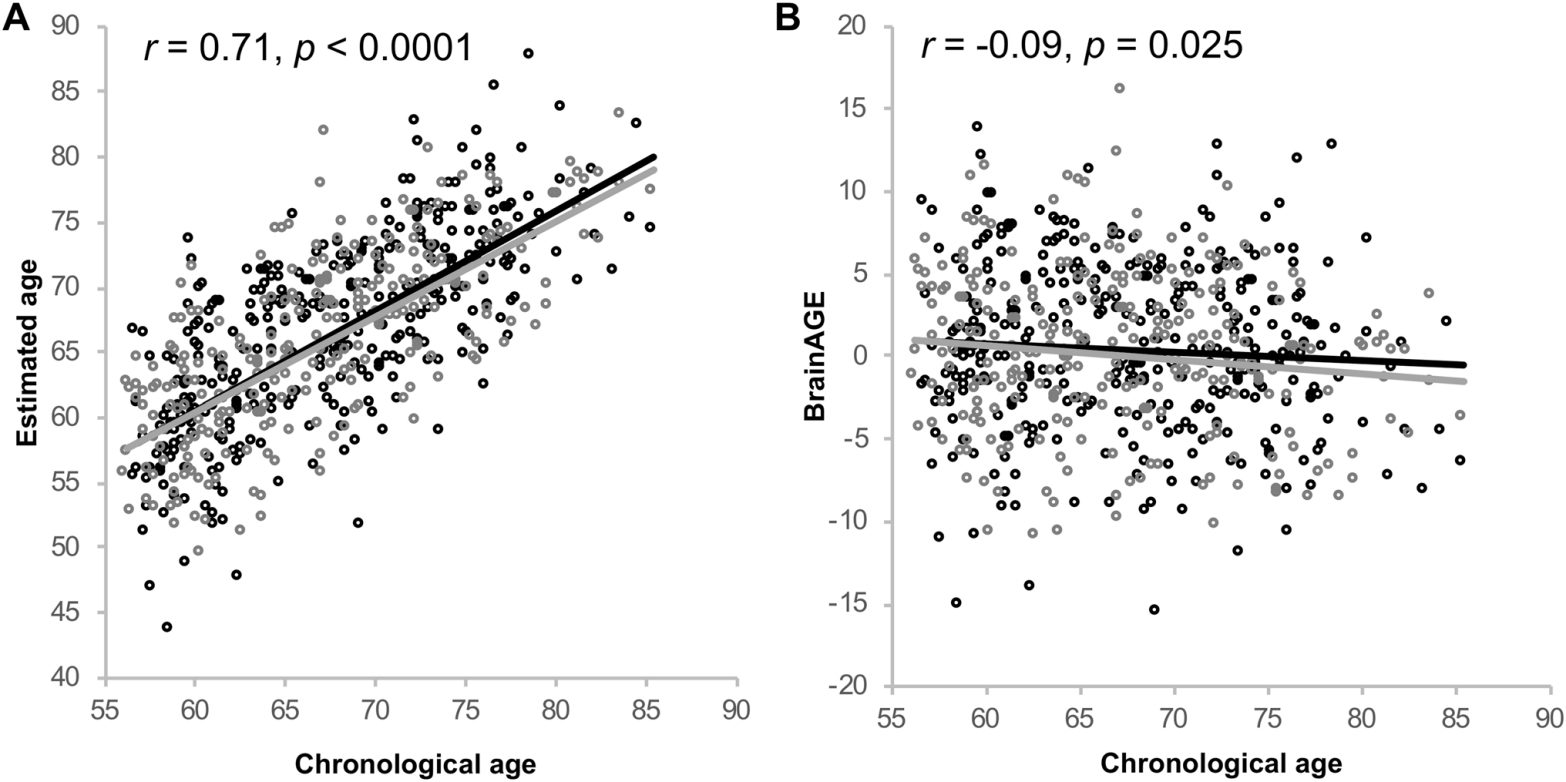
Scatter plots for the older subsample of *n* = 622 used in the lifestyle analyses. **a** Correlation between estimated and chronological age was significant with *r* = 0.71 (*p* < 0.0001). **b** Correlation between chronological age and BrainAGE was *r* = − 0.09 (*p* = 0.025). Black dots represent men, dark-grey dots represent women. Sex-specific correlation values can be found in Table 2

Within the older subsample (*n* = 622), which we used for our main analysis of the association between lifestyle and BrainAGE, mean BrainAGE was 0.23 years (SD = 4.96) with a maximum positive deviation between chronological and estimated age of  + 15.92 years (brains appearing older compared to their chronological age) and a maximum negative deviation of – 15.67 years (brains appearing younger compared to their chronological age). MAE was 3.97 (SD = 2.99) years, and did not differ between the two sexes [*T*(2, 620) = 0.20, *p* = 0.839]. The regression of estimated age on chronological age explained up to 52% of the variance (Table 1). The correlation between chronological and estimated age was *r* = 0.714 (*p* = 0.0001, Table 2, Fig. 3a), whereas the correlation between chronological age and BrainAGE was *r* =− 0.10 (*p* = 0.025, Fig. 3b). Hence, the older the participants are, the lower were the BrainAGE scores.

### Relation between covariates and BrainAGE in the older adult sample

In the between-subject MANCOVA, there was no significant difference between female and male participants in chronological age, *F*(1,621) = 1.55, *p* = 0.213, *η*^2^ = 0.002, nor in BrainAGE, *F*(1, 621) = 1.35, *p* = 0.246, *η*^2^ = 0.002. Mean estimated age was 1.28 years lower for females than for males with *F*(1,621) = 3.98, *p* = 0.046, *η*^2^ = 0.005 (Table 1). In the between-subjects ANCOVA, males showed a higher educational level than females [*F*(1,621) = 39.50, *p* < 0.0001, *η*^2^ = 0.06]. No correlation between BrainAGE and education was found (*p* = 0.937), even when stratifying the analyses for the two sexes (Table 3). Physical and mental well-being was high (mean = 78.66, max possible: 100, Suppl. Table 1a). On average participants completed the DemTect test with an overall score of 14.8 points (out of 18). Only eight participants met the criteria for being at risk for dementia (Suppl. Table 1b). We excluded these participants, as well as participants, who did not complete the DemTect test in the models where cognitive status was introduced to see if results stayed stable.

**Table 3:** Correlations between ISCED and chronological age, estimated age and BrainAGE

Spearman correlations	Chronological age	Estimated age	BrainAGE
ISCED			
Whole subsample (*n* = 622)	*ρ* = − 0.14, *p* < 0.001	*ρ* = 0.13, *p* = 0.001	*ρ* = − 0.03, *p* = 0.937
Male (*n* = 350)	*ρ* = − 0.11, *p* = 0.040	*ρ* = 0.12, *p* = 0.028	*ρ* = − 0.06, *p* = 0.251
Female (*n* = 272)	*ρ* = − 0.16, *p* = 0.008	*ρ* = − 0.13, *p* = 0.029	*ρ* = 0.02, *p* = 0.705

Spearman correlations between general level of education as measured by ISCED with chronological age, estimated age, and BrainAGE for the older subsample of lifestyle analyses

*ρ* Spearman correlation coefficient

### Main analyses of lifestyle and BrainAGE

#### Combined lifestyle risk

In our first analysis of lifestyle, we investigated the association between combined lifestyle risk and BrainAGE. Table 4 shows descriptive statistics for all individual lifestyle variables as well as for the combined lifestyle risk score. Mean combined lifestyle risk was − 1.02 (*SD* = 2.1), reflecting a rather protective behavior within the selected older subsample of 1000BRAINS.

**Table 4:** Descriptive statistics of lifestyle variables

		Min	Max	Mean	SD
Pack-years	Total sample (male, female)	0.00 (0.00, 0.00)	204.00 (204.00, 123.00)	13.61 (16.27, 1.19)	21.73 (24.37, 17.21)
Alcohol consumption	Total subsample	0.00	198.50	11.25	19.87
	(male, female)	(0.00, 0.00)	(198.50, 79.40)	(15.85, 5.33)	(23.86, 10.46)
Physical activity	Total subsample	0.00	196.00	14.32	20.94
	(male, female)	(0.00, 0.00)	(196.00, 189.00)	(14.69, 13.86)	(21.07, 20.81)
Social integration	Total subsample	3.00	53.00	12.99	6.40
	(male, female)	(3.00, 4.00)	(53.00, 44.00)	(13.19, 12.72)	(6.53, 6.24)
Combined lifestyle risk	Total subsample	− 9.60	10.36	− 1.02	2.06
	(male, female)	(− 9.60, − 9.08)	(10.36, 4.96)	(0.72, 1.40)	(2.19, 1.81)

Descriptive statistics for all four individual lifestlye variables, as well as the combined lifestlye risk score for the total older subsample (*n* = 622), as well as for older males (*n* = 350) and older females (*n* = 272)

We now investigated how well combined lifestyle risk predicted BrainAGE in the multiple linear regressions implementing all three approaches to deal with chronological age [approach 1 including chronological age as predictor, 2 ignoring age and 3 examining the age-corrected residuals]. Please also see Table 5 for all regression statistics.

**Table 5:** Linear regression statistics examining combined lifestyle risk for all three approaches

	Whole subsample (*n* = 622)			Male (*n* = 349)			Female (*n* = 272)		
	Includes “sex” as a covariate			No covariate			No covariate		
	1	2	3	1	2	3	1	2	3
Risk score	*β* = 0.182 *T* = 4.543 *p* < 0.0001	*β* = 0.183 *T* = 4.566 *p* < 0.0001	*β* = 0.182 *T* = 4.546 *p* < 0.0001	*β* = 0.193 *T* = 3.658 *p* < 0.0003	*β* = 0.193 *T* = 3.656 *p* < 0.0003	*β* = 0.193 *T* = 3.664 *p* < 0.0003	*β* = 0.157 *T* = 2.630 *p* < 0.009	*β* = 0.162 T = 2.700 *p* = 0.007	*β* = 0.158 *T* = 2.633 *p* = 0.009
*F*	8.77****	10.72****	10.73****	7.46****	13.36****	13.42****	5.50**	7.29**	6.93**
*R*^2^	0.041 (0.032)	0.034 (0.033)	0.034 (0.032)	0.041 (0.037)	0.037 (0.037)	0.037 (0.037)	0.039 (0.025)	0.026 (0.026)	0.025 (0.025)
MAE (SD)	3.93 (2.85)	3.93 (2.85)	3.93 (2.85)	4.01 (2.93)	4.01 (2.95)	4.01 (2.93)	3.82 (2.76)	3.84 (2.78)	3.82 (2.76)

	Sex, education, BMI, DemTect (*n* = 595)			Education, BMI, DemTect (*n* = 334)			Education, BMI, DemTect (*n* = 262)		
Risk score	*β* = 0.158 *T* = 3.733 *p* < 0.0002	*β* = 0.164 *T* = 3.925 *p* < 0.0001	*β* = 0.159 *T* = 3.808 *p* < 0.0002	*β* = 0.162 *T* = 2.912 *p* = 0.004	*β* = 0.164 *T* = 2.944 *p* = 0.003	*β* = 0.162 *T* = 2.92 *p* = 0.004	*β* = 0.138 *T* = 2.246 *p* = 0.026	*β* = 0.146 *T* = 2.392 *p* = 0.017	*β* = 0.139 *T* = 2.266 *p* = 0.024
*F*	5.26***	4.707****	4.855***	2.676*	2.822*	2.855*	4.074***	2.967	3.4943**
*R*^2^	0.049 (0.023)	0.038 (0.025)	0.04 (0.024)	0.039 (0.025)	0.032 (0.026)	0.034 (0.025)	0.074 (0.019)	0.060 (0.023)	0.058 (0.020)
MAE (SD)	3.90 (2.90)	3.92 (2.92)	3.90 (2.99)	3.90 (2.95)	4.00 (2.96)	3.99 (2.95)	3.73 (2.84)	3.75 (2.88)	3.73 (2.84)

****p < 0.0001, ***p <  = 0.001, **p < 0.01, *p < 0.05; approach 1 included chronological age as a predictor in the regression models of lifestyle on BrainAGE, 2 did not include chronological age within the model and 3 used the residuals of BrainAGE after chronological age was regressed out of BrainAGE. If the DemTect test was added as covariate, only participants, who completed the DemTect and reached a score > 8 were included in that specific analysis. *R*^2^ in brackets is the amount of *R*^2^ only explained by the combined lifestyle risk score, when already adjusted for all covariates, if present. *p* values of the *β*-coefficients are rounded to three decimals

**Fig. 4:**
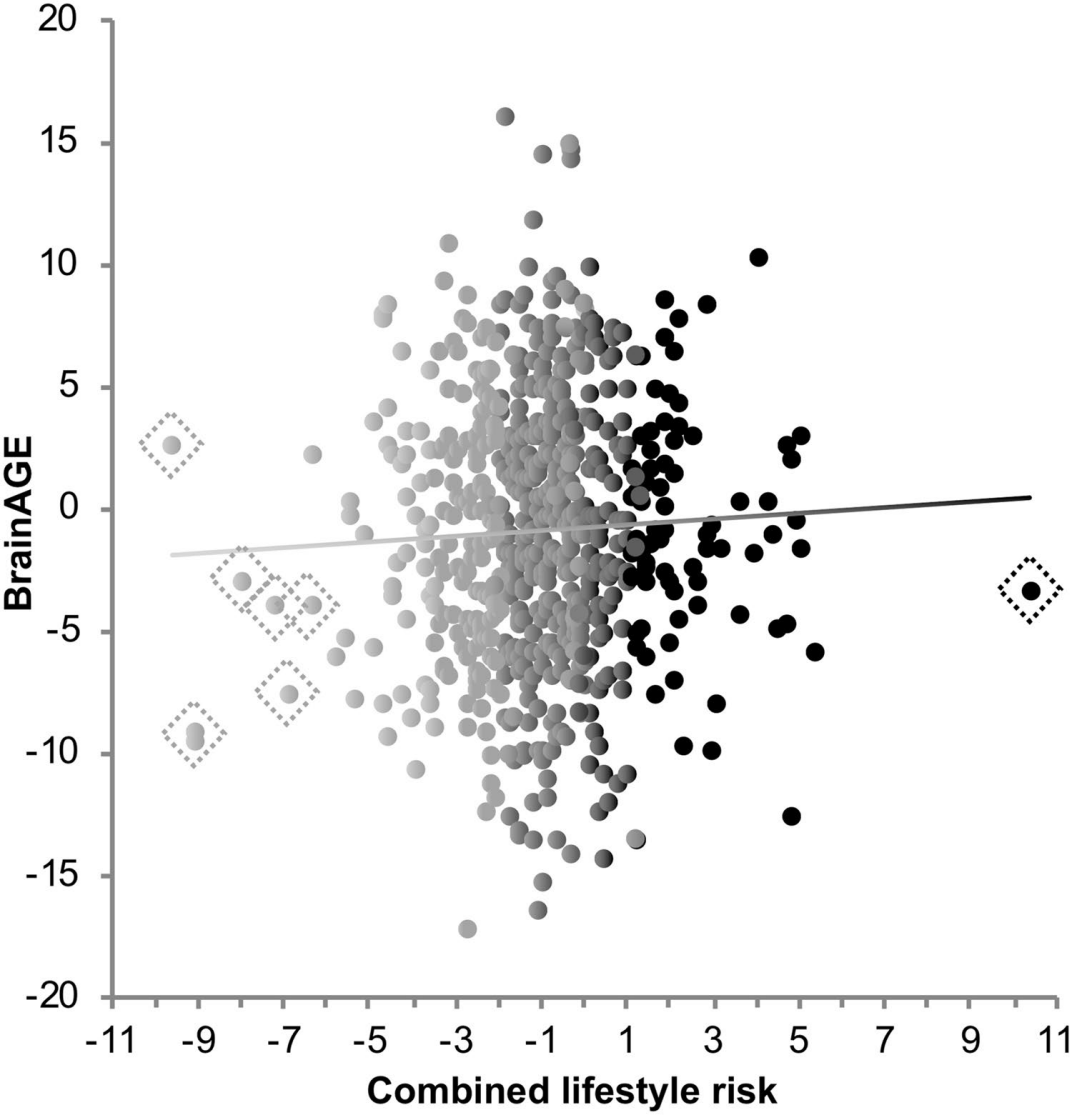
Correlation between combined lifestyle risk and BrainAGE. Higher combined lifestyle risk was associated with higher BrainAGE. The color spectrum depicts the increase in lifestyle risk from protective (light grey) to balanced (dark grey) to more risky (black) behaviour. Values are raw values to represent the general association, which was more specifically examined using different adjustements for chronological age in the three different approaches 1, 2 and 3

Combined lifestyle risk significantly predicted BrainAGE in all three age approaches using sex as covariate: Higher combined lifestyle risk was predictive of higher BrainAGE, with a regression coefficient of *β* = 0.182 to *β* = 0.183, all *p* < 0.001. These results remained stable, even after outlier exclusion (all *p* < 0.001, Fig. 4; outlier marked with diamond shapes, Table 5), introducing education *(*all *p* < 0.001), as well as cognitive status (DemTect) and BMI (all *p* < 0.001) as covariates. All of these associations were significant at *p*_adjusted_ ≈ 0.003. Within the fully adjusted model, the combined lifestyle risk score predicted 2.3–2.5% of the variance in BrainAGE, with a MAE of about 3.93 years in all approaches.

#### Individual lifestyle variables

**Fig. 5:**
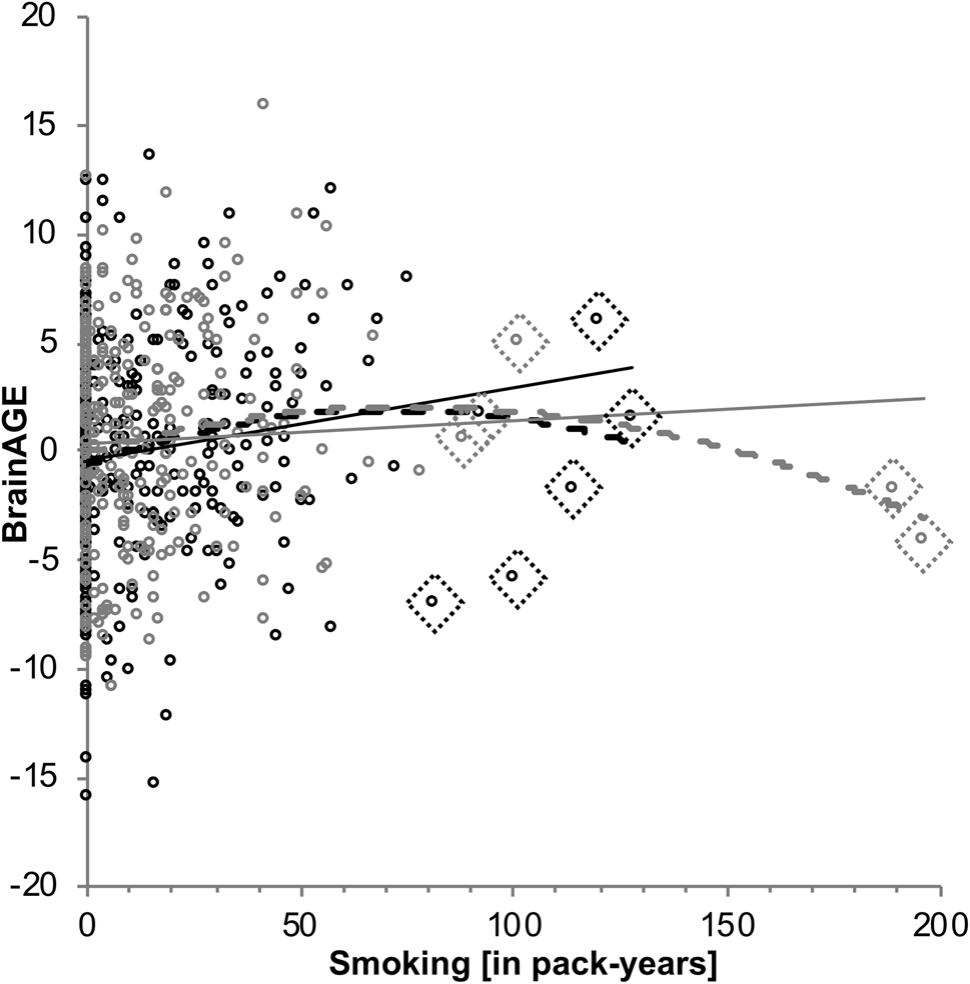
Association between smoking and BrainAGE. Higher BrainAGE was associated with higher amount of smoking. Black dots represent men, light-grey dots represent women. The interaction between sex and amount of smoking on BrainAGE was not significant. Dashed lines represent the fit of a quadratic function into the data. Values are raw values to represent the general association, which was more specifically examined using different adjustments for chronological age in the three different approaches 1, 2 and 3

In the next step, we examined which individual lifestyle variables were predictive of BrainAGE by including all four individual lifestyle variables into a multiple linear regression as explanatory variables, while correcting for sex. This was done for all three approaches. Overall, the model significantly predicted BrainAGE, *F*(6,615) = 5.14–5.17, all *p* =  < 0.001, *R*^2^ = 0.040–0.048. Neither alcohol consumption (*β* = 0.049–0.053, all *p* > 0.197, *R*^2^ = 0.002), nor social integration (*β* = − 0.054–0.055, all *p* > 0.169, *R*^2^ = 0.002) significantly predicted BrainAGE (Suppl. Table 3). But a higher amount of smoking predicted higher BrainAGE, with *β* = 0.136–0.140, all *p* =  < 0.001. Smoking was still predictive of BrainAGE after outlier correction (*β* = 0.170–0.175, all *p* < 0.0001; Fig. 4; 10 outlier marked with diamond shapes), as well as adding educational level (*β* = 0.169–0.175, all *p* < 0.0001), and cognitive status (*β* = 0.173–0.181, all *p* < 0.0001) as covariates. In this fully adjusted model, pack-years of smoking predicted 2.9–3.2% of the variance in BrainAGE with a MAE of 3.88–3.90 years (Table 6) and remained significant as well after outlier exclusion (Fig. 5, marked with diamond shapes, *β* = − 0.147–0.149, all *p* < 0.001). Higher physical activity remained significantly associated with lower BrainAGE after correction for all three additional variables, i.e., BMI, education and cognitive status in all three approaches (*β* = − 0.144–0.146, all *p* < 0.001) above *p*_adjusted_ ≈ 0.003. It was predictive of 2.0–2.1% of variance in BrainAGE with a MAE of 3.90–3.93.

**Table 6:** Prediction statistics from linear regressions examining pack-years for all three approaches

	Whole subsample, outlier corrected *n* = 622			Male, outlier corrected *n* = 342			Female, outlier corrected *n* = 270		
	Includes sex as a covariate			No covariate			No covariate		
	1	2	3	1	2	3	1	2	3
Pack-years	*β* = 0.170 *T* = 4.235 *p* < 0.0001	*β* = 0.175 *T* = 4.355 *p* < 0.0001	*β* = 0.171 *T* = 4.241 *p* < 0.0001	*β* = 0.166 *T* = 3.103 *p* = 0.002	*β* = 0.164 *T* = 3.065 *p* = 0.002	*β* = 0.166 *T* = 3.104 *p* = 0.002	*β* = 0.142 *T* = 2.330 *p* = 0.021	*β* = 0.160 *T* = 2.659 *p* = 0.008	*β* = 0.140 *T* = 2.312 *p* = 0.022
*F*	8.150***	9.872****	9.487****	5.766**	9.395**	9.636**	4.903**	7.070**	5.346**
*R*^2^	0.039	0.031	0.03	0.033	0.027	0.027	0.035	0.026	0.02
MAE (SD)	3.90 (2.91)	3.91 (2.93)	3.90 (2.91)	4.02 (2.98)	4.02 (3.01)	4.02 (2.98)	3.78 (2.84)	3.79 (2.86)	3.79 (2.83)

	Sex, education, DemTect (*n* = 593)			Education, DemTect (*n* = 331)			Education, DemTect (*n* = 263)		
Pack-years	*β* = 0.173 *T* = 4.211 *p* < 0.0001	*β* = 0.181 *T* = 4.397 *p* < 0.0001	*β* = 0.175 *T* = 4.265 *p* < 0.0001	*β* = 0.168 *T* = 3.044 *p* = 0.003	*β* = 0.168 *T* = 3.040 *p* = 0.003	*β* = 0.168 *T* = 3.049 *p* = 0.002	*β* = 0.148 *T* = 2.418 *p* = 0.016	*β* = 0.166 *T* = 2.747 *p* = 0.006	*β* = 0.147 *T* = 2.424 *p* = 0.016
*F*	5.899****	5.742***	5.764***	3.052*	3.133*	3.195*	4.754**	5.330***	4.805**
*R*^2^	0.048 (0.029)	0.038 (0.032)	0.038 (0.029)	0.036 (0.027)	0.028 (0.027)	0.028 (0.028)	0.031 (0.025)	0.058 (0.028)	0.053 (0.022)
MAE (SD)	3.88 (2.95)	3.90 (2.96)	3.88 (2.95)	4.01 (3.00)	4.02 (3.03)	4.01 (3.01)	3.73 (2.86)	3.73 (2.89)	3.73 (2.85)

****p < 0.0001, ***p <  = 0.001, **p < 0.01, *p < 0.05; approach 1 included chronological age as a predictor in the regression models of lifestyle on BrainAGE, 2 did not include chronological age within the model and 3 used the residuals of BrainAGE after chronological age was regressed out of BrainAGE. If the DemTect test was added as covariate, only participants, who completed the DemTect and reached a score > 8 were included in that specific analysis. *R*^*2*^ in brackets is the amount of *R*^2^ only explained by pack-years of smoking, when already adjusted for all covariates, if present. *p* values of the *β-*coefficients are rounded to four decimals

#### Sex differences in the association between lifestyle and BrainAGE

##### Combined lifestyle risk

The between-subjects ANCOVA did not show a significant interaction between sex and the combined lifestyle risk score on BrainAGE [*F*(4, 617) = 0.18, *p* = 0.672], even after outlier correction [*F*(4, 617) = 0.37, *p* = 0.545]. Nonetheless, we were interested in investigating sex differences further in exploratory analyses. After stratifying the group by the two sexes to account for overall sex differences in BrainAGE, higher combined lifestyle risk was still associated with higher BrainAGE in all age approaches in both males (all *β* = 0.193, all *p* < 0.001, *R*^2^ = 0.037) and females (*β* = 0.158–0.162, *p* = 0.007–0.009, *R*^2^ = 0.025–0.026). Neither introducing education, nor BMI and cognitive status as additional covariates changed these associations (Table 5), nor outlier exclusion (Fig. 4, outliers marked with diamond shapes). In the fully adjusted model the combined lifestyle risk score predicted 2.5–2.6% of variance (MAE = 3.99–4.00 years) in males and 1.9–2.3% of variance in BrainAGE (MAE = 3.73–3.75) in females. The associations in females were not significant after multiple comparison correction (*p*_adjusted_ ≈ 0.003), though.

##### Individual lifestyle variables

We did not find any significant interaction effects of sex and the individual lifestyle variables on BrainAGE in the between-subject MANCOVA. After stratifying for the two sexes, smoking and physical activity showed significant effects (Tables 6, 7). A higher amount of smoking still significantly predicted higher BrainAGE for male [*β* = 0.164–0.166, all *p* = 0.002, *R*^2^ = 0.027–0.033 for the whole model], as well as for female participants [*β* = 0.140–0.160, *p* = 0.008–0.022, *R*^2^ = 0.035 for the whole model]. After outlier correction (Table 6, Fig. 5, outliers marked with diamond shapes) and adding education and cognitive status as covariates (model 3) higher pack-years predicted 2.7–2.8% of variance in BrainAGE (MAE = 4.01 years) in males and 2.2–2.8% (MAE = 3.85–3.89 years) in females. However, the associations in females did not survive multiple comparison correction. The association between higher physical activity and lower BrainAGE, though, remained significant for male participants [*β* = − 0.186 to − 0.188, all *p* =  < 0.001, *R*^2^ = 0.035–0.040 for the whole model], but not for female participants [*β* = − 0.089 to − 0.097, all *p* > 0.11, *R*^2^ = 0.008–0.026 for the whole model]. In males, this association was significant despite outlier correction, adding education, BMI and cognitive status as covariates and multiple comparison correction (Table 7) with physical activity predicting 3.4–3.6% of the variance in BrainAGE (MAE = 4.01–4.03). In females, physical activity predicted only up to 0.8% of the variance in BrainAGE (MAE = 3.70–3.75) in the fully adjusted model, which was not significant.

**Table 7:** Prediction statistics from linear regressions examining physical activity for all three approaches

	Whole subsample, outlier corrected *n* = 613			Male, outlier corrected *n* = 345			Female, outlier corrected *n* = 268		
	Includes sex as a covariate			No covariate			No covariate		
	1	2	3	1	2	3	1	2	3
Physical activity	*β* = − 0.148 *T* = − 3.712 *p* < 0.0003	*β* = − 0.147 *T* = − 3.683 *p* < 0.0003	*β* = − 0.149 *T* = − 3.714 *p* < 0.0003	*β* = − 0.186 *T* = − 3.501 *p* = 0.001	*β* = − 0.188 *T* = − 3.541 *p* < 0.0005	*β* = − 0.186 *T* = − 3.506 *p* = 0.001	*β* = − 0.097 *T* = − 1.601 *p* = 0.111	*β* = − 0.089 *T* = − 1.457 *p* = 0.146	*β* = − 0.097 *T* = − 1.592 *p* = 0.113
*F*	6.72***	7.068***	7.276***	7.047***	12.541***	12.291***	3.53*	2.123	2.535
*R*^2^	0.03	0.023	0.023	0.04	0.035	0.035	0.026	0.008	0.009
MAE (SD)	3.93 (2.86)	3.95(2.88)	3.94 (2.86)	4.03 (2.84)	4.04 (2.85)	4.03 (2.84)	3.82 (2.86)	3.84 (2.90)	3.82 (2.85)

	Sex, education, BMI, DemTect (n = 594)			Education, BMI, DemTect (n = 333)			Education, BMI, DemTect (n = 261)		
Physical activity	*β* = − 0.144 *T* = − 3.516 *p* < 0.0005	*β* = − 0.146 *T* = − 3.546 *p* = 0.0005	*β* = − 0.146 *T* = − 3.531 *p* = 0.0005	*β* = − 0.187 *T* = − 3.392 *p* = 0.009	*β* = − 0.192 *T* = − 3.475 *p* = 0.001	*β* = − 0.188 *T* = − 3.416 *p* = 0.001	*β* = − 0.088 *T* = − 1.433 *p* = 0.153	*β* = − 0.084 *T* = − 1.347 *p* = 0.179	*β* = − 0.080 *T* = − 1.422 *p* = 0.156
*F*	4.690***	3.916**	4.237***	3.139**	3.375**	3.318*	3.695**	3.20*	3.405**
*R*^2^	0.046 (0.020)	0.032 (0.021)	0.035 (0.021)	0.046 (0.034)	0.040 (0.036)	0.039 (0.034)	0.068 (0.008)	0.048 (0.007)	0.051 (0.008)
*MAE (SD)*	3.90 (2.89)	3.93 (2.91)	3.90 (2.90)	4.01 (2.86)	4.03 (2.87)	4.01 (2.86)	3.70 (2.92)	3.75 (2.95)	3.70 (2.92)

****p < 0.0001, ***p <  = 0.001, **p < 0.01, *p < 0.05; approach 1 included chronological age as a predictor in the regression models of lifestyle on BrainAGE, 2 did not include chronological age within the model and 3 used the residuals of BrainAGE after chronological age was regressed out of BrainAGE. If the DemTect was added as covariate, only participants, who completed the DemTect and reached a score > 8 were included in that specific analysis. *R*^2^ in brackets is the amount of *R*^2^ only explained by physical activity, when already adjusted for all covariates, if present. *p* values of the β-coefficients are rounded to three decimals

#### Exploration of non-linear lifestyle effects

**Fig. 6:**
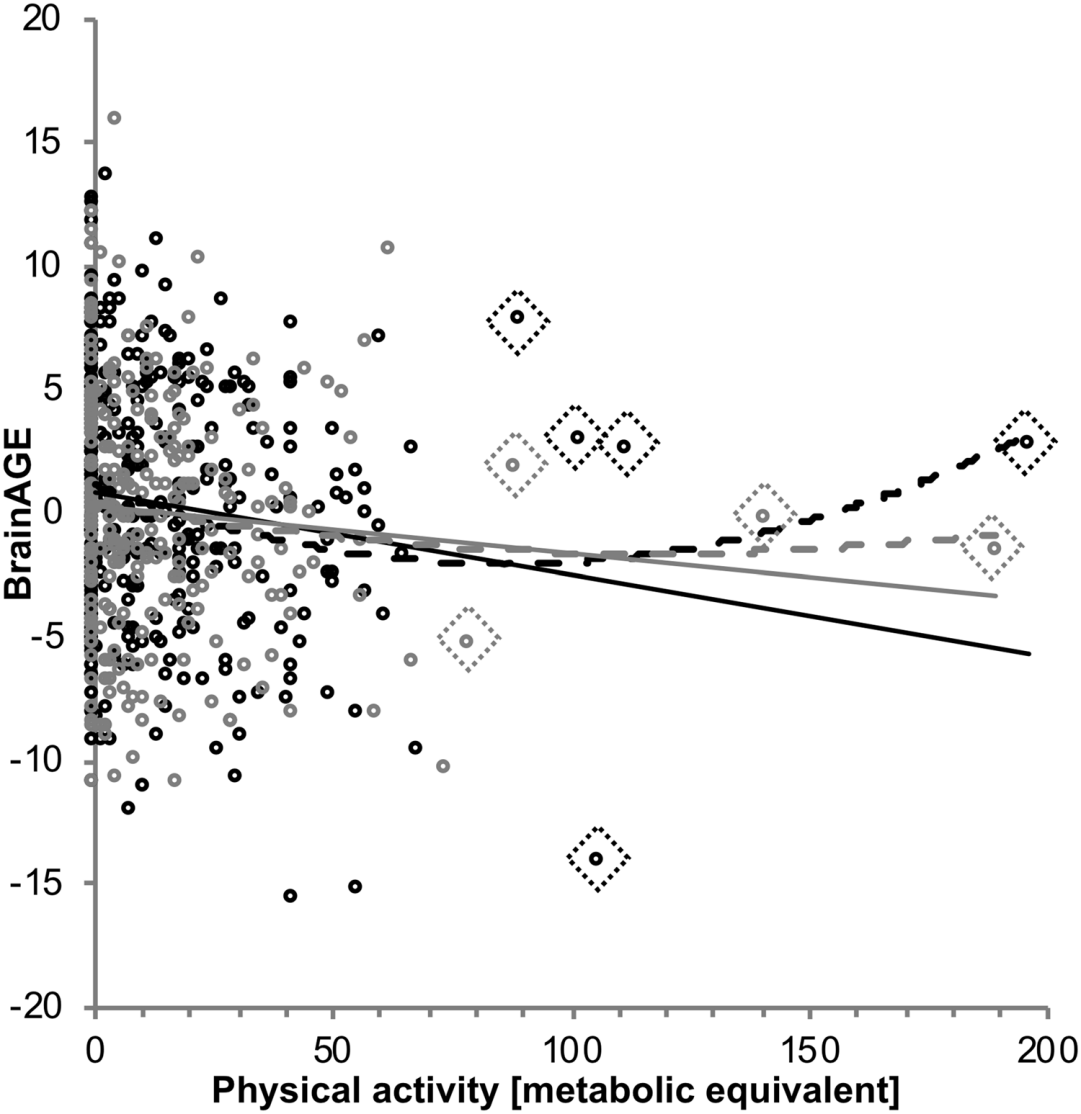
Association between physical activity and BrainAGE. Lower BrainAGE was associated with higher physical activity as measured with the metabolic equivalent. Light-grey dots represent female, black dots represent male participants. The interaction effect between sex and physical activity on BrainAGE was not significant. The dashed lines represent quadratic trends. Regression lines were significant for males, whereas they were not significant for females. Values are raw values to represent the general association, which was more specifically examined using different adjustments for chronological age in the three different approaches 1, 2 and 3

Finally, we tested for quadratic effects of the combined lifestyle risk score and individual lifestyle variables on BrainAGE. Lifestyle variables that were used as regressors in the curve fitting process were already corrected for sex, education, and, in case of the combined lifestyle risk score and physical activity, BMI. Therefore, the *R*^2^ values within this estimation differ from those in the purely linear regressions. In the following section, the subscript “q” indicates that the regressors belong to the quadratic function (Fig. 6).

##### Combined lifestyle risk

Adding a quadratic term to the linear regression of BrainAGE on combined lifestyle risk did not result in a significantly better fit than the linear regressions, with both functions explaining 2.4–3.0% of the variance and the quadratic term itself not being significant, which was also true after outlier correction and for each approach. After splitting the sample by sex, there was no difference between the linear and quadratic fit for male (*R*^2^_linear_ = 0.026–0.030, *R*^2^_quadratic_ = 0.030–0.035; all *p* > 0.225), and for female participants (*R*^2^_linear_ = 0.020–0.022, *R*^2^_quadratic_ = 0.020–0.022; *p* > 0.882), which was also true after outlier correction. All regression statistics can be found in Suppl. Table 6.

##### Individual lifestyle variables

Adding the quadratic term to the linear model describing the association between smoking and BrainAGE resulted in marginally higher explained variance (*R*^2^_linear_ = 0.022–0.023, *R*^2^_quadratic_ = 0.023–0.025), while the quadratic term itself was not significant (all *p* > 0.269). This was also true in male participants only (*R*^2^_linear_ = 0.021–0.022, *R*^2^_quadratic_ = 0.24–0.026; all *p* > 0.273, Suppl. Table 7). After adding the quadratic term to the linear model for female participants, the model was no longer predictive of BrainAGE (Suppl. Table 7).

Regarding the association between physical activity and BrainAGE, the quadratic term showed an additional effect on BrainAGE (*R*^*2*^_linear_ = 0.011–0.012; *R*^*2*^_quadratic_ = 0.019–0.020; *β*_*2q*_ = 0.126–0.135, *p* < 0.034), which was no longer significant after outlier correction (all *p* > 0.765, Suppl. Table 8). In male participants, including the quadratic term did also result in a slightly better model fit (all *R*^2^_linear_ = 0.018; *R*^2^_quadratic_ = 0.035–0.036, all *p* < 0.017), but this effect as well as the better model fit disappeared after outlier correction (all *p* > 0.0552, Suppl. Table 8). In female participants, neither the quadratic nor the linear model showed a significant association between physical activity and BrainAGE with even lower estimates of explained variance (*R*^2^ = 0.004–0.005) than in the linear regressions only.

##### Quantification of lifestyle effects

For one increase in the combined risk score (risk score = 1), BrainAGE was estimated at 4.92 months older in addition to the effect of sex, chronological age, education and cognitive status (approach 1), which the beta values had already been corrected for (BrainAGE = 1.747 + (− 0.90) × sex + (− 0.76) × age + (− 0.029) × education + 0.077 × BMI + 0.146 × DemTect + 0.412 × riskscore). Without chronological age (approach 2), BrainAGE was estimated at 5.16 months older and using the age-corrected residuals BrainAGE was estimated 5.04 months older. For each pack-year of cigarettes, BrainAGE was estimated at 0.6 months older in approach 1 (BrainAGE = 2.91 + (− 0.229) × sex + (− 0.07) × age + (− 0.032) × education + 0.164 × DemTect + 0.5 × pack-years), which was slightly higher with 0.62 months in approach 2 and and the same for approach 3. Finally, we calculated an increase in BrainAGE of 0.55 months per pack-year in males and 0.56 months per pack-year in females (BrainAGE_male_ = 4.283 + (− 0.07) × age + (− 0.015) × education + 0.019 × DemTect + 0.46 × pack-years; BrainAGE_female_ = − 0.326 + (− 0.07) × age + (− 0.052) × education + 0.361 × DemTect + 0.47 × pack-years). For males this estimation was the same for all three approaches. For females the increase in BrainAGE was estimated higher in approach 2 with 0.63 month, but the same for approach 3. Comparing never, moderate and severe smokers revealed, that the brains of severe smokers (BrainAGE = 1.61) appeared significantly older than those of never (BrainAGE = − 0.05) and moderate (BrainAGE = − 0.03) smokers, but that there was no significant difference between never-smokers and moderate smokers in average BrainAGE.

For each metabolic equivalent that was expended per week, BrainAGE was estimated 0.55 month younger when including chronological age in approach 1 (BrainAGE = 3.445 + (− 0.492 × sex + (− 0.088) × age + (− 0.056) × education + 0.056 × BMI + 0.176 × DemTect + (− 0.046) × physical activity). When not including chronological age (approach 2), the estimation was slightly higher with 0.56 months of BrainAGE, but this vanished when chronological age was regressed out before estimation (approach 3). Finally, we calculated a decrease in BrainAGE of 0.70 months per metabolic equivalent in males and 0.35 months per metabolic equivalent in females (BrainAGE_male_ = 4.135 + (− 0.06) × age + (− 0.058) + (− 0.039) × education + 0.042 × BMI + 0.012 × DemTect + (− 0.058) × physical activity; BrainAGE_female_ = − 0.115 + (− 0.105) × age + (− 0.058) × education + 0.078 × BMI + 0.383 × DemTect + (− 0.029) × physical activity). This was the same, when regressing chronological age out of the BrainAGE score before estimation (approach 3) in both sexes. When not including chronological age (approach 2), the estimation of BrainAGE increase was almost identical with 0.72 months in males, and 0.34 months in females.

#### Power analysis

With *n* = 622 participants, an alpha level of 0.003 and number of predictors = 5 our power to detect an expected small (Cohen 2013) effect size of 0.05 (Karama, Bug and Head) was 0.99 for the regression of BrainAGE onto combined lifestyle risk within the whole subsample. With an expected effect size of 0.04 for pack-years of smoking our power was 0.97. With an expected effect size of 0.07 for physical activity, our power was 0.99.

## Discussion

The present study showed that lifestyle habits contribute to differences in brain aging in a cohort of “normal” aging older adults and gives promising insights into why people even without neurodegenerative diseases age so differently. We used two approaches: We used a novel lifestyle risk score (Bittner et al. 2019) combining different lifestyle variables into one value, while additionally investigating each lifestyle habit alone. In addition, we examined differences in brain structure using BrainAGE as a meaningful imaging biomarker (Franke et al. 2012, 2014; Gaser et al. 2013; Lowe et al. 2016), which showed a very good performance in our sample (Cole and Franke 2017). Further, the results of our subsequent analyses may provide first hints at sex differences in the association between lifestyle and BrainAGE that are often not examined in studies on lifestyle-associated differences in the brain, e.g., in terms of smoking (Karama et al. 2015), alcohol consumption (Vergara et al. 2017) and physical activity (Kramer and Colcombe 2018).

### Associations between combined lifestyle risk and BrainAGE

Prior studies focused mostly on the effect of single lifestyle variables on the brain, considering co-occurrences of various lifestyle behaviors as nuisance factors rather than as effects of interest. In contrast, the current study considered four different lifestyle behaviors as a combined concept (Bittner et al. 2019) to examine if lifestyle explains variability in BrainAGE and which lifestyle behaviors contribute the most to this association. Regarding a phenotype as complex and multidimensional as lifestyle, it is reasonable to assume that one specific behavior can only account for parts of the variance in brain aging, providing necessity for investigation of different variables together (Bittner et al. 2019; Floel et al. 2008; Vergara et al. 2017). Considering several behaviors as well as composite scores seem to provide a better prediction of differences between older adults, e.g., in verbal memory, than the individual measures alone (Floel et al. 2008). Comparable approaches have also been used in imaging genetics, where polygenic risk scores (several genetic markers aggregated into one score) can explain more variability in neurological diseases and brain phenotypes than individual genetic markers alone (Dudbridge 2013; Harrison et al. 2016; Torkamani et al. 2018; Ursini et al. 2018). Considering the seed and soil model of neurocognitive disorders, it may be expected that a soil that promotes neuronal alterations gets even more toxic, the more risk factors come together (McDonough and Allen 2019). Therefore, we hypothesized that our combined lifestyle risk score would predict more variance than each single behavior alone.

To test this hypothesis, we conducted three different approaches: approach 1 including chronological age as a predictor, approach 2 ignoring chronological age and approach 3 regressing chronological age out of the BrainAGE score before the analyses. In general, the results of those three models were in agreement. Approach 1 was predictive of the highest amount of variance in BrainAGE, likely, since chronological age was still associated to this outcome, and therefore, contributed a small amount of prediction. The amount of variance in BrainAGE explained by the different lifestyle variables, i.e., the combined lifestyle risk score, physical activity and pack-years of smoking was greatest in approach 2. Therefore, the quantification in months of BrainAGE was also highest in this approach, but these differences were marginal. This is most likely due to the fact, that if no correction for chronological age is made, there is some variance in BrainAGE which would be explained by chronological age usually, but which will now be predicted by lifestyle behavior, which is slightly confounded with chronological age. The prediction accuracy measured in MAE was comparable between the approaches though. Before regressing chronological age out of BrainAGE in approach 3 it was still associated to BrainAGE with *p* = 0.028, but only explaining 0.8% of the variance in the whole sample. This was not true in male (*R*^2^ = 0.005; *p* = 0.209), but in female (*R*^2^ = 0.015, *p* = 0.047) participants, which was expectable since a small correlation between chronological age and BrainAGE is visible in females (Fig. 2b). In fact, the association between chronological age and BrainAGE in the whole sample may be driven by the female participants.

However, from a theoretical perspective, it seems contra-intuitive to include chronological age into a model to predict an outcome, which is related to age itself, favoring approach 2. Since BrainAGE was still—at least in females—associated to chronological age, not correcting BrainAGE for chronological age would be slightly inaccurate though, from a statistical perspective. In our view, approach 3 seems, therefore, to be the most appropriate approach, since it does not include chronological age as a predictor (to predict BrainAGE), but the residual variance of chronological age, which is statistically there, is regressed out, nevertheless. However, there is no conclusive solution to this issue in the present literature (Smith et al. 2019).

As hypothesized, we found a lifestyle-dependent acceleration of structural brain aging, where higher lifestyle risk predicted higher BrainAGE scores, thus older looking brains. This observation is particularly important, since it helps explain a significant proportion of the large interindividual variability in structural brain aging of older adults (Dickie et al. 2013), which cannot be accounted for by age, sex, education or clinical markers, such as BMI or uric acid (Arenaza-Urquijo et al. 2015; Christie et al. 2017; Eavani et al. 2018; Fjell and Walhovd 2010; Franke et al. 2014; Jagust 2013). In the present sample, the combined lifestyle risk score predicted 2.4% (approach 3) of the variance beyond sex, education, BMI and cognitive status, which was comparable to the amount predicted by physical activity (2.1%) and smoking (2.9%), also in approach 3. This is comparable to the amount of explained variance reported for lifestyle behaviors or health markers in other large epidemiological studies (Franke et al. 2014; Jockwitz et al. 2019; Miller et al. 2016) and also within reasonable range for a large sample from a population-based cohort (Bzdok and Ioannidis 2019). It is important to note, that even though each variable only predicted a small proportion of variance, this variance is calculated after correction for those factors usually showing a large influence on brain structure: age, sex, education and general cognitive status. Even after correction, the general association between riskier lifestyle and older looking brains remained stable. However, other influences, such as genetic variations may have an additional impact on age-related differences in brain structure and age predictions (Ning et al. 2020; Smith et al. 2020).

In a former study, individual lifestyle variables did not show a significant effect on cortical surface measures, whereas the combined lifestyle risk score did (Bittner et al. 2019). However, it is important to note, that the former study was a vertex-wise whole brain approach, sensitive to lifestyle-related regional differences, whereas the present study examined the association between lifestyle and BrainAGE, reflecting the multidimensional pattern of aging aggregated into one marker. Importantly, we quantified the effect of combined lifestyle risk on structural brain aging in terms of years. This was inspired by Franke et al. (2014), who estimated mean BrainAGE in a “risky” and a “healthy” group in terms of clinical markers, such as BMI or uric acid. Instead of quantifying group differences in terms of years, as done by Franke et al. (2014), we estimated the linear increase in BrainAGE for each increase in lifestyle risk of the specific variable. In consequence, with each increase in combined lifestyle risk, brains appear 5.04 months older than the “normal” age-related difference in brain structure, which the statistical model corrected for. In comparison, brains appear 0.6 months older with each pack-year and 0.55 months younger with each increase in metabolic equivalent (MET) per week (with, e.g., 4 MET reflecting one hour of 10-mph bicycling). Hence, the combined lifestyle risk score explained more than 3 months in Brain AGE “in addition” to smoking in pack-years, as hypothesized. Therefore, the combined lifestyle risk score may have higher explanatory power, presumably via consideration of over-additive and interacting effects between the individual factors when quantifying the harmful and protective effects of lifestyle. Considering different behaviors of interest simultaneously may thus be a fruitful way to explain additional variance in brain aging by investigating their cumulative effects.

### Associations between individual lifestyle variables and BrainAGE

Investigating the four lifestyle behaviors individually revealed more smoking and lower physical activity to be the strongest contributors to the prediction capacity of lifestyle risk on BrainAGE.

#### Smoking

One of the compelling results of the current study were the negative effects of smoking on the aged brain quantified in months, which were also not corroborated after correcting for education or cognitive status. Prior studies already hinted at an association between smoking and changes in GM. Regionally lower GM volume and density for smokers compared to non-smoking control participants have been reported in the prefrontal cortex and the cerebellum (Brody et al. 2004), the posterior cingulum, precuneus, right thalamus, and bilateral frontal cortex (Almeida et al. 2008), as well as the substantia nigra (Gallinat et al. 2006). Importantly, all of these studies had a fairly small sample size that either included younger adults only [*n* = 45, age range 22.4–38.3 years, Gallinat et al. (2006)], older adults only [*n* = 78, age range 71.6–78.9 years; Almeida et al. (2008)], or a large age range [*n* = 36, 21–65 years, Brody et al. (2004)]. In addition, results on the association of smoking with other brain metrics are quite heterogenous. Higher numbers of white matter hyperintensities (Longstreth et al. 2005), lower microstructural integrity (Gons et al. 2011), or infarcts (Howard et al. 1998) in smokers compared to non-smokers were reported. With population-based cohort imaging available, the sample sizes have substantially increased (Bamberg et al. 2015; Caspers et al. 2014; Miller et al. 2016; Van Essen et al. 2013), thus increasing generalizability of results to the general population. For example, Karama et al. (2015) showed that smoking was associated with widespread cortical thinning in a sample of 504 older adults particularly in prefrontal cortex, mostly omitting primary sensory areas. Still, none of the studies provided a quantification of the effect of smoking on the brain. We were able to predict an increase of 0.36 months of BrainAGE with each pack-year, with 2.9% of BrainAGE being predicted just by smoking (approach 3). Translating this result to our older adult study sample taking into account the average smoking behavior of 13.61 pack-years, an overall increase of 4.9 years of BrainAGE (13.61 × 0.36 month of BrainAGE) only by smoking can be stated.

There was a high variance in BrainAGE in individuals, who never smoked (139 female, 114 male, Fig. 4). Within this group, BrainAGE scores were very high, as well as very low, aggregating to a mean BrainAGE of almost zero. This finding is comparable to the considerable variance in cortical thickness in the rarely smoking participants observed by Karama et al. (2015). In the current study, the more the participants smoked, the stronger was the relationship between higher pack-years and higher BrainAGE, suggesting that this effect was mostly driven by high lifetime smoking (Fig. 4). This was also revealed when comparing never, moderate and severe smokers, where the brains of severe smokers (BrainAGE = 1.61) appeared significantly older than those of never (BrainAGE = − 0.05) and moderate (BrainAGE = − 0.03) smokers. It is particularly important to note that this observation cannot be translated simply to the assumption that rare smoking has no effect on the brain. Rather, rare smoking may manifest in other metrics for healthy brain aging, even if alterations in brain structure would not be present: For example, in our previous study, we found no association between smoking and cortical surface measures in older adults. Instead, more smoking was associated with higher resting-state functional connectivity (RSFC), which may be a compensation mechanism for accelerated brain aging (Bittner et al. 2019). In addition, activity differences in task-based fMRI (Lawrence et al. 2002; Tanabe et al. 2011), as well as receptor differences between smokers and non-smokers (Feduccia et al. 2012; Mukhin et al. 2008) were described. Studies on RSFC in relation to long-term effects of smoking and not acute effects of nicotine are rather rare. It may thus be of particular interest to investigate general differences in brain function, e.g., in RSFC, associated with light smoking (Janes et al. 2012; Pariyadath et al. 2014; Zhou et al. 2017), even though light smoking seems not to be heavily associated to differences in brain structure.

The underlying mechanisms driving the association between smoking and changes in brain structure are still unclear. Smoking could potentially act via atherosclerotic processes, which may impact the aging brain and thus accelerate brain aging (Freund et al. 1993; Mucha et al. 2006; Prescott et al. 1998; Pujades-Rodriguez et al. 2015). Possibly, the measured increase in BrainAGE might also be attributable to the direct toxic effects of tobacco smoke onto the cerebro-vascular system, which includes oxidative stress within the cells and results in apoptosis (Swan and Lessov-Schlaggar 2007). However, since BrainAGE takes the whole GM volume of an individual into account, drawing inferences about any molecular mechanisms or disentangle regional differences that drive the association between stronger smoking and accelerated brain aging as reflected by higher BrainAGE remains for future studies. Still, our results provide evidence, that smoking may be one of the detrimental factors contributing to an unfavorable soil, which then together with additional risk factors, promotes alterations within the brain or accelerated already existing processes of structural decline.

##### Sex differences in the associations between smoking and BrainAGE

Most prior studies assessing the effect of smoking on brain structure did not examine sex differences or the interaction of sex and smoking (Almeida et al. 2008; Brody et al. 2004; Gallinat et al. 2006; Karama et al. 2015; Longstreth et al. 2005). To our knowledge, there are only two studies addressing this issue, showing that structural differences associated to smoking may regionally differ depending on sex (Duriez et al. 2014; Franklin et al. 2014). After examining lifestyle effects in the whole study sample, we addressed this issue and found no interaction between sex and smoking, hinting at a comparable direction and strength of association in both sexes. With imaging research focusing more on sex differences (Franke et al. 2014; Gur and Gur 2017; Ritchie et al. 2018; Ruigrok et al. 2014; Wierenga et al. 2018), it may nevertheless be of interest to identify lifestyle behaviors that differentially affect male and female brains, such that interventions that slow or delay manifestations of aging can be tailored for sex.

#### Physical activity and BrainAGE

The protective effect of physical activity on GM volume has been discussed to be regionally specific [see review by Erickson et al. (2014)]. Our results support that higher physical activity is predictive of lower BrainAGE, thus younger looking brains. Physical activity, therefore, does not only seem to affect specific brain regions, but also the multidimensional pattern of brain aging itself. Most previous studies comprised intervention trainings, where training was systematic, regular, and highly controlled (Erickson et al. 2014). The present study adds to this by demonstrating that higher physical activity is associated with decelerated brain aging (lower BrainAGE scores) in a large sample of “normal” aging older adults using a comprehensive, epidemiologically motivated measurement of physical activity, i.e., the metabolic equivalent (Ainsworth et al. 2000; Bus et al. 2011; Floel et al. 2010; Milanovic et al. 2013; Pierce et al. 2007; Ruscheweyh et al. 2011; Wagner et al. 2012). This measurement is drawn from self-reports that summarize all sorts of sports older adults engage in and is likely to reflect the average daily physical activity, in contrast to highly controlled intervention settings. The present association between self-reported physical activity and BrainAGE is, therefore, not as strong as reported effects of fitness training on, e.g., the hippocampus (Erickson et al. 2011), but is likely reflecting a natural, and therefore, more generalizable relationship.

Several mechanisms how higher physical activity or fitness levels may act protectively on the aging brain have been discussed, such as the upregulation of neurotrophic factors, including brain-derived-neurotrophic factor [BDNF, (de Melo Coelho et al. 2013; Neeper et al. 1996; Piepmeier and Etnier 2015) Ruscheweyh et al. 2011] and granulocyte-colony stimulating factor (G-CSF; Flöel et al. 2010), which significantly impact synaptic efficacy, neuronal connectivity, and use-dependent plasticity. Here, use-dependent plasticity may play a crucial role in the sense that physical activity as one kind of training would lead to better preservation of those brain structures needed to perform the activity engaged in (Bittner et al. 2019; Colcombe et al. 2003; Vaynman and Gomez-Pinilla 2005; Vaynman et al. 2004). Several studies have shown that training-induced preservation or even adaptation of brain regions is possible in adults (Churchill et al. 2002; Draganski et al. 2004; Erickson et al. 2011; Kramer and Erickson 2007) or older adults in particular (Boyke et al. 2008). Nevertheless, other studies have discussed, that the most important factor for preservation of brain structure is effortful learning (Shors 2014). During physical activity, both processes are possible, but could not be controlled in the epidemiological setting of the present study. However, physical activity is widely known for its benefitting effects on the general health of the human organism (World Health Organization 2019). Possibly enhancing the resilience of the organism, as well as higher neuronal integrity (Engeroff et al. 2019), it may, therefore, contribute to a more protective soil, where pathological processes may have lesser effect (McDonough and Allen 2019). Future studies examining specific mechanisms are needed, though.

##### Sex differences in the associations between physical activity and BrainAGE

Most previous studies on lifestyle did not examine interaction effects of sex, as done in classic psychological research or specifically conducted sex-stratified analyses, as done in epidemiological research (Erickson et al. 2014; Floel et al. 2010, 2008; Ho et al. 2011). Yet, a recent review concluded that the sex proportion in physical activity intervention studies may impact the effect sizes (Kramer and Colcombe 2018). A potential reason may be expression of BDNF and its effect on physical activity, which has been shown to differ between the sexes in mice with lower expression in females (Venezia et al. 2016). Further, estrogens or hormone replacement therapy seem to be related to levels of neurotrophins such as BDNF (Garcia-Segura et al. 2000) and longer periods of hormone therapy may corroborate the positive effect of high physical activity in women (Erickson et al. 2007). There may also be further differences between the two sexes that co-occur with physical activity, such as dietary habits (Kramer and Colcombe 2018), the specific kind of activity (Churchill et al. 2002; Colcombe et al. 2003; Floel et al. 2010; Hayes et al. 2013; Kramer and Colcombe 2018), as well as differences in metabolism (Burd et al. 2009; Wu and O'Sullivan 2011) to be considered in future studies. Therefore, we specifically addressed sex differences in subsequent analyses. We did not find a significant interaction effect of sex and physical activity on BrainAGE. Post hoc stratification of the sample for the two sexes revealed that physical activity was significantly predictive for BrainAGE in male (*p* = 0.005), but not in female participants (*p* = 0.169). This, however, may be due to the smaller proportion of females in the whole sample. Hence, it is possible, that the effect is present in females, but even larger sample sizes are needed to detect it in females as well (i.e., more power). The statistical significance is non-conclusive, hence. The picture gets more unambiguous, when inspecting the amount of predicted variance in BrainAGE (*R*^2^). Here, physical activity was predictive of 3.4% of variance in BrainAGE in males, but of a strikingly different 0.8% of variance in females. This hints at physical activity contributing more to BrainAGE prediction in males.

Interestingly, the (in-sample) prediction accuracy measured in MAE for any of the models examining lifestyle was highest in females. In females, the lowest MAE was 3.70 years in the fully adjusted model examining physical activity. For comparison, the lowest MAE in males was MAE = 3.99 years (fully adjusted model for the combined lifestyle risk score) and in the whole subsample MAE = 3.88 years (fully adjusted model examining pack-years) showing that factors contributing to prediction capacity may differ between males and females (Jiang et al. 2020).

Interestingly, the most accurate model for the prediction of BrainAGE in females was also the model, where physical activity did not significantly contribute to prediction, but which was mostly driven by cognitive status (fully adjusted model examining physical activity). Note that all models including cognitive status (measured using the DemTect) predicted a relatively high proportion of variance in BrainAGE in females (up to 7.4% in approach (i) in combination with the combined lifestyle risk score). This proportion was considerably higher in females than in males (3.9% explained variance within the same model). Hence, cognitive status may in females be a more important mediator when it comes to the prediction of structural brain aging from lifestyle behavior, whereas lifestyle behavior may contribute more to prediction in males.

Thus, the current study found hints for sex differences within this association, but larger samples are needed. Taken together, higher physical activity seems to be one lifestyle behavior that may be predictive of decelerated brain aging, in line with previous studies. As Kramer and Colcombe (2018) state in their recent review, it can be of great help to disentangle the association between physical activity and BrainAGE to facilitate, e.g., large exercise programs within the communities.

#### Alcohol consumption and social integration

We did not find an association between BrainAGE and social integration or alcohol consumption. Regional differences in brain structure associated to social integration, as well as alcohol consumption could be present, but might not have been identifiable with the specific approach of the current study. Several explanations might hold for these observations. To date, the number of studies investigating social integration in relation to structural brain decline in older adults is relatively small (for a recent review, see Anaturk et al. 2018). In addition, most studies report effects for composite measurements of cognitive and social activities, which do not clearly differentiate between social and cognitive components (Gow et al. 2012; Hafsteinsdottir et al. 2012; Vaughan et al. 2014). Further, composite measures for social activities when investigated in combination with additional lifestyle behaviors (Bittner et al. 2019) have been assessed. Therefore, future studies could shed light on effects of social activities with low cognitive versus high cognitive demands to answer the question whether the cognitive or the social component of social integration contributes to brain reserve, the amount to which age-related GM loss can be tolerated without showing deficiencies (Stern, 2012). In addition, we used a quantitative measurement of social integration. Studies have shown that older adults engage in relationships with a focus on quality rather than quantity (Carstensen et al. 1999). Hence, future studies would be needed to address the association between quality of relationships and brain aging. Further, differences in brain structure related to social integration may be regional or subtle (James et al. 2012), which also seems to be the case for alcohol consumption (Topiwala et al. 2017). Even though accelerated brain aging has been shown in patients with alcoholism (Pfefferbaum et al. 1995), differences related to alcohol consumption in the normal population may not be as strong or only identifiable if several risk behaviors co-occur (Bittner et al. 2019). Further, alcohol consumption may affect other brain parameters earlier such as WM lesions (den Heijer et al. 2004) or RSFC (Vergara et al. 2017). In addition, effects of alcohol consumption may also be non-linear (Mukamal et al. 2001), which we could not identify in the present study, but might be interesting for future studies to further investigate into.

### Strengths and limitations

Strengths of the present study include the large sample size, the older age range of the sample, and the use of BrainAGE as a state-of-the art imaging biomarker. By conceptualizing brain aging in this biomarker, the multivariate dataset and the statistical analyses could be reformulated into intuitive and straightforward results, i.e., the quantification of lifestyle effects in meaningful months of additional brain age.

The present study has a cross-sectional design which does not allow conclusions about directionality of effects. Even without a longitudinal design, though, our approach hints at individuals with higher risk for accelerated brain aging: Comparing the apparent image-based age of individuals’ brains enables a measure of whether a participant’s brain appears to be older (or younger) than the average age-matched data of the sample and captures deviation from expected, i.e., typical development (Kaufmann et al. 2019). For future studies, regional specificity, i.e., disentangling the relevance of different regions for machine-learning-based brain age prediction would be highly desirable (Cole and Franke 2017). Further, investigating the relevance of different brain features, e.g., functional activation patterns, as well as functional and structural connectivity, will be interesting in the future. As BrainAGE enables the identification of individuals at higher risk, together with the straightforward statistics and intuitive quantification of lifestyle effects in meaningful months, it still provides a useful framework to capture relevant aspects of variability in structural brain aging and to examine the high variability in brain reserve (Stern, 2017).

Further, it is important to mention that, based on established approaches in epidemiological research (Schmermund et al. 2002), the lifestyle variables included in our combined lifestyle risk score were measured using different time windows (e.g., physical activity was assessed for the last 4 weeks, smoking as the number of cigarettes smoked over the whole lifetime). Assessments that refer to specifically defined short time frames (e.g., a month, a week) seem to be more reliable indicators of long-term behavior than self-reports referring to longer time frames, e.g., a whole year (Del Boca and Darkes 2003).

In addition, all lifestyle habits were assessed using self-reports, which makes it impossible to rule out memory effects or social desirability bias. Self-report measurements have nevertheless been shown to be valid and reliable (Del Boca and Darkes 2003) and thus suitable in such an epidemiological cohort setting.

## Conclusion

Higher lifestyle risk, represented by a combined lifestyle risk score, contributes to accelerated brain aging as revealed by BrainAGE, a meaningful imaging biomarker. Higher lifetime smoking, as well as lower physical activity contributed most to this association. We found hints at a stronger relation between physical activity and BrainAGE for males than females, but future studies are needed to further evaluate the potentially differential relevance of this influencing factor for brain health between the sexes. Further, more research is needed to elucidate the relation between alcohol consumption and brain structure, as well as social integration and brain health, e.g., by disentangling the cognitive and social components. In summary, lifestyle seems to be a fruitful target for identifying behaviors that may contribute to a more resilient organism, and therefore, slow neuronal changes and related or resulting cognitive impairment. Future studies are warranted to examine the underlying mechanisms. Considering co-occurrences between several lifestyle behaviors as effects of interests, rather than as a nuisance may enable us to better understand individual trajectories of brain aging in the older population and why people age differently.

## Supplementary Information

Below is the link to the electronic supplementary material.

Supplementary file1 (XLSX 44 KB)

## Abbreviation

ISCED: International standard classification of education
